# Geographical characterisation of British urban form and function using the spatial signatures framework

**DOI:** 10.1038/s41597-022-01640-8

**Published:** 2022-09-07

**Authors:** Martin Fleischmann, Daniel Arribas-Bel

**Affiliations:** 1 Geographic Data Science Lab, Department of Geography and Planning, University of Liverpool, Roxby Building, 74 Bedford St S, Liverpool, L69 7ZT UK; 2 The Alan Turing Institute, British Library, 96 Euston Road, London, England NW1 2DB UK

**Keywords:** Geography, Society

## Abstract

The spatial arrangement of the building blocks that make up cities matters to understand the rules directing their dynamics. Our study outlines the development of the national open-source classification of space according to its form and function into a single typology. We create a bespoke granular spatial unit, the enclosed tessellation, and measure characters capturing its form and function within a relevant spatial context. Using K-Means clustering of individual enclosed tessellation cells, we generate a classification of space for the whole of Great Britain. Contiguous enclosed tessellation cells belonging to the same class are merged forming spatial signature geometries and their typology. We identify 16 distinct types of spatial signatures stretching from wild countryside, through various kinds of suburbia to types denoting urban centres according to their regional importance. The open data product presented here has the potential to serve as boundary delineation for other researchers interested in urban environments and policymakers looking for a unique perspective on cities and their structure.

## Abstract (summary)

**Table:** 

Measurement(s)	Urban environment
Technology Type(s)	Urban morphometrics, geodemographics
Sample Characteristic - Environment	city

## Background & Summary

**Fig. 1:**
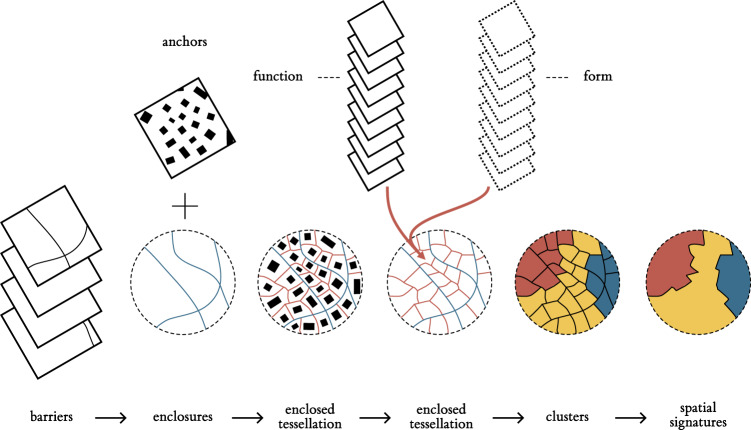
Diagram illustrating the sequential steps leading to the delineation of spatial signatures. From a series of enclosing components, to enclosures, enclosed tessellation (ET), the addition of form and function characters to ET cells, and the development of spatial signatures.

**Fig. 2:**
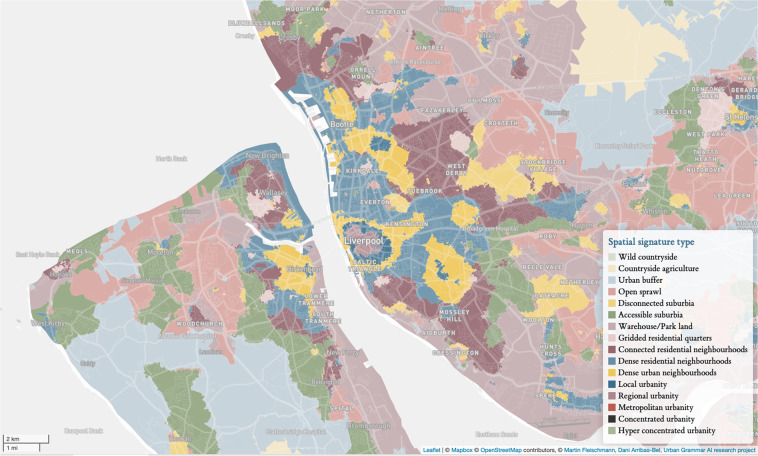
Illustration of a classification of spatial signatures in Liverpool and Birkenhead area, in the north west of England.

How the building blocks that make up cities are spatially arranged is worth quantifying and understanding. By “building blocks”, we mean both the activities and agents that inhabit cities, as well as the (infra)structure that supports them. The former can be conceptualised as *urban function*, while the latter falls under the study of *urban form*. Understanding urban form and function is important for two main reasons. First, the combination of both *encodes* rich information about the history, character and evolution of cities. For example, the shape and properties of the street network encode the technology of the time (e.g., automobile); while the degree of mix in land uses can reflect cultural values. Second, the spatial pattern of urban form and function also acts as a frame that *influences* a variety of outcomes, from economic productivity to socio-economic cohesion to environmental sustainability. In this paper, we use the Spatial Signatures framework^1,2^, which develops a “characterisation of space based on form and function designed to understand urban environments”^1^. Spatial signatures are theory-informed, data-driven computable classes that describe the form and function of a consistent patch of geography. Figure 1 presents an overview of the development of a spatial signature classification. We build a series of enclosures that we combine with building footprints to further subdivide geographical space into what we call enclosed tessellation cells (ETCs). We then attach form and function characters to each of these subdivisions, and use those to group them into consistent and differentiated classes we call signatures. Each phase is expanded in detail in the next section. We introduce an open data product (ODP^3^) containing a classification of spatial signatures for Great Britain (illustrated in a Fig. 2). In doing so, we provide an analysis-ready layer that brings together urban form and function consistently, in detail, and at national scale. To the best of our knowledge, this is the first dataset capturing urban form and function published both with a degree of detail and scale as ours. Our results are based on the analysis of more than 14 million of ETCs, to each of which we attach more than 300 characters capturing a wide range of aspects relating to urban form and function. We provide access to both granular geographical boundaries of the delineated spatial signatures as well as measurements for each character at the signature level. The ODP also includes a web map that allows exploration without any technical requirement other than a web browser, and we have open sourced all the code, including details on the computational backend. The uniqueness of our ODP makes it challenging to set up a technical validation as a comparison with existing datasets. Nevertheless, we relate our signatures to a few well-established data products that capture each a subset of the form and function dimensions we consider. Our results are encouraging in that they show broad agreement in expected areas, but also highlight aspects that can only be discovered when considering form and function in tandem. The approach and outputs presented bring several benefits to a range of stakeholders interested in cities. This spatial signatures ODP provides insight generated from detailed, comprehensive and computationally intensive data analysis and presents it in a way that is easy to access, work with and integrate into larger projects. Together with the importance of form and function discussed above, we anticipate the output will be relevant to both academic researchers as well as policymakers and practitioners. As a framework, the spatial signatures provide a flexible yet generalisable way to understand, characterise and quantify urban form and function. One way to understand our results is as an application to Great Britain of a more general approach to quantitatively characterise the spatial dimension of cities. As such, our conceptual approach can be applied in many more local contexts and regions beyond Great Britain. It is true that Great Britain currently represents an unusual case in that it is specially “data dense”, with a large variety of open data that may not be readily available in other parts of the world. However, given form and function reinforce each other, spatial signatures are designed to be robust to variations in the specific data sources used, and two different classifications do not need to be based on exactly the same data to be useful. At the same time, we note that the combination of volunteered geographic information (e.g., OpenStreetMap) and technologies such as modern satellites and artificial intelligence are filling many of these gaps very rapidly, and we anticipate near-future developments that will make the implementation of classifications such as the one presented here possible in almost any (urban) area of the planet. In this sense, our ODP (data, code, and methodology) can be a useful illustration for researchers and practitioners who, even if not specifically interested in the British use case, would like to implement a similar approach on their own. As illustration of potential applications, we provide two. The spatial signatures may be used to delineate types of (origin and destination) locations in mobility analysis, that could unveil patterns of commuting or migration in situations like the COVID-19 pandemic. A second application may focus directly on supporting policy on inequalities. For example the spatial signatures can underpin analysis on equality of access to services and amenities within the UKs Levelling Up agenda^4^, using them to target areas based on their signature type, since they will share key structural components. It is important to note we do not expect signatures to focus on a single aspect of urban environment as, for example, Local Climate Zones^5^ do with climate, but instead on a wider range of uses due to their inclusion of both form and function and a data driven nature reflecting the specific place rather than abstract conceptual classes. In this respect, we hope the present paper serves not only to document our own work but to inspire future efforts aimed at urban form and function.

## Methods

The method of identification of spatial signatures consists of three top-level steps. First, we delineate a spatial unit of analysis that reflects the structure of urban phenomena on a very granular level. Then we characterise each of them according to form and function, capturing the nature of each unit and its spatial context. Finally, we use cluster analysis to derive a typology of our spatial units that, once combined into contiguous areas, forms a typology of spatial signatures.

### Spatial unit

**Fig. 3:**
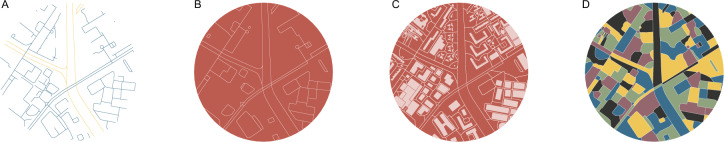
Diagram illustrating the sequential steps leading to the delineation of enclosed tessellation. From a series of enclosing components, where blue are streets and yellow river banks (**A**), to enclosures (**B**), incorporation of buildings as anchors (**C**) to final tessellation cells (**D**).

The first major methodological decision relates to the definition of the spatial unit. An ideal candidate needs to reflect space in a granular manner, and we argue it should fulfil three conditions. First, it should be *indivisible*, meaning that any subdivision would result in a unit that is incapable of capturing the nature of urban form and function. Second, it needs to be *internally consistent* - it should always reflect only a single signature type. Last, it should be geographically *exhaustive*, covering the entirety of the study area. Spatial units used in literature can be split into three groups. One is using administrative boundaries like city regions^6^, wards or census output areas^7^, that are convenient to obtain and can be easily linked to auxiliary data. However, those rarely reflect the morphological composition of urban space and, in some cases, may even “obscure morphologic reality”^8^. At the same time, most of them are divisible, and larger units are not always internally consistent. Another group is based on arbitrary uniform grids linked either to spatial indexing methods like H3^9^ or Ordnance Survey National Grid, or to ancillary data of remote sensing or other origins like a WorldPop grid^10^. Grids however cannot be considered internally consistent as they do not consider the underlying structure of the landscape. Finally, urban morphology studies tend to use morphological elements as street segments^11^, blocks^12^, buildings^13^ or plots^14^ as units of analysis. Some of those could be seen as indivisible and internally consistent, but since they are largely based on built-up fabric, they are not exhaustive. For example, in areas without any building or street, there is no spatial unit to work with. Plots could be theoretically considered as exhaustive, consistent and indivisible, but there is no accepted conceptual definition and unified geometric representation^15^. We are, therefore, proposing an application of an alternative spatial unit called *enclosed tessellation cell* (ETC), defined as “the portion of space that results from growing a morphological tessellation within an enclosure delineated by a series of natural or built barriers identified from the literature on urban form, function and perception”^1^. ETCs follow the morphological tradition in that it is based on the physical elements of an environment but overcome the drawbacks of conventionally used units. Its geometry is generated in the three steps illustrated in a Fig. 3. First, a set of features representing physical barriers subdividing space, in our case composed of the street network, railways, rivers and a coastline, is combined, generating a layer of boundaries (3 A). These then partition space into smaller enclosed geometries called *enclosures* (3 B), which can be very granular or very coarse depending on the geographic context. In dense city centres where a single enclosure represents a single block is a high frequency of small enclosures. At the same time, in the countryside, this approach leads to very few large enclosures as their delimiters are far away from each other. Enclosures are then combined with building footprints (3 B), which act as anchors in space and potentially subdivide enclosures into enclosed tessellation cells using the morphological tessellation algorithm^16^ (3 D), a polygon-based adaptation of Voronoi tessellation. The resulting geometries are indivisible as they contain, at most, a single anchor building, internally consistent due to their granularity and link to morphological elements composing urban fabric, and geographically exhaustive as they cover an entire area limited by specified boundaries. In our ODP for Great Britain, street networks are extracted from OS Open Roads datasets^17^ representing simplified road centrelines cleaned of underground road segments. Railways are retrieved from OS OpenMap - Local^18^ (“RailwayTrack” layer) which captures surface railway tracks. Rivers are extracted from OS OpenRivers^19^ representing river network of GB as centrelines, and a coastline is retrieved from OS Strategi®^20^, capturing coastline as a continuous line geometry. Building geometry is extracted, again, from OS OpenMap - Local (“Building” layer) and represents generalised building footprint polygons. Note that the dataset does not distinguish between individual buildings when they are adjacent (e.g. perimeter block composed of multiple buildings is represented by a single polygon).

### Characterisation of space

Spatial signatures capture the character of the built and unbuilt environment based on two components - form and function. Each of them is quantified at the level of individual ETCs using methods appropriate for each specific dataset. While form is described using urban morphometrics (i.e. quantitative analysis of urban form)^21^, function is a composite of a variety of data inputs. We outline each component with a bit more detail below.

#### Form

**Fig. 4:**
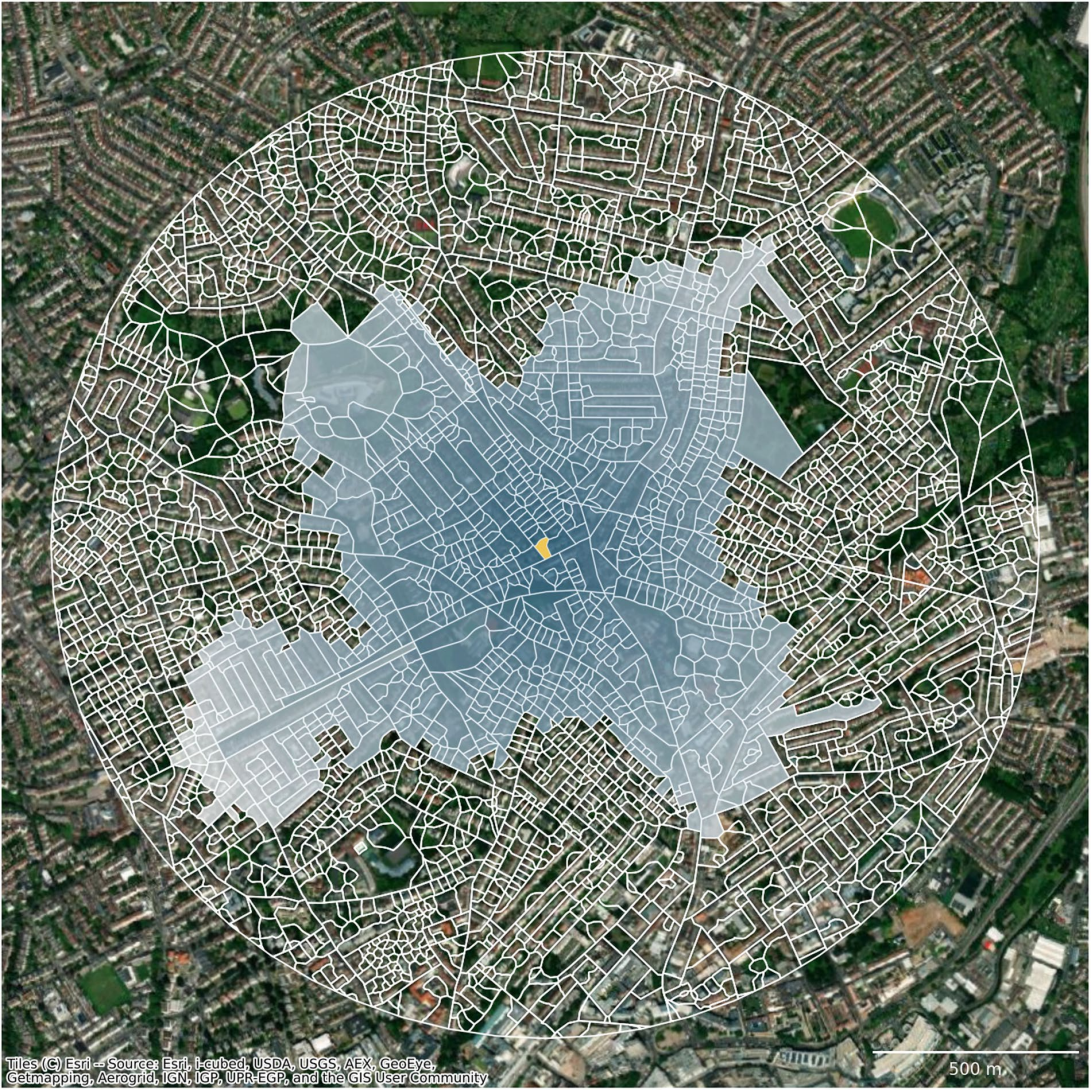
Illustration of a definition of spatial context used to capture the distribution of values around each ET cell. For the yellow ET cell in the middle, we propose to define a neighbourhood of 10 topological steps on the tessellation and weight the importance of each cell within such an area by inverse distance between poles of inaccessibility of each cell.

Morphometric characterisation of urban form is based on the numerical description of four elements capturing the built environment - buildings, streets, ETCs, and enclosures - and reflects their patterns based on six categories of characters: dimensions, shapes, spatial distribution, intensity, connectivity and diversity^22^. Each element is considered across different scales, from the measurement of individual geometries, to relations of neighbouring geometries, to a graph-based analysis of the street network. The combination of elements, categories and scales results in a set of 59 individual morphometric characters listed in the Tables 1 and 2. The selection builds on the principles outlined by^21^ and later explored by^23^, both following the rules derived by^24^. The gist is to include as many characters present in literature as is feasible, while minimising potential collinearity and limiting redundancy of information. That can be caused by capturing the same phenomena, like a specific aspect of the shape of a building, using multiple characters. Note that the characters that are statistically correlated but capture different concepts are kept as such information reflects the nature of urban form and thus increases the robustness of the method. However, measuring individual characters is not enough to understand the predominant spatial patterns. For some types of urban environment, high heterogeneity is not uncommon. This means that using, for example, areas of building footprints would, in most cases, result in largely discontinuous clusters that do not capture the pattern within an area. Therefore, we represent each of the morphometric characters using three summary variables reflecting statistical distributions of measured data within a spatial context of each ETC. Context is defined as tenth order of contiguity computed across the mesh composed of contiguous ETCs as illustrated in Fig. 4. Furthermore, each value is weighted by the inverse distance between so-called poles of inaccessibility (defined as a centre of a maximum inscribed circle) of each ETC. Three proxy variables then capture the first, the second and the third quartile of the resulting weighted distribution. Such a characterisation can capture the contextual tendency of each morphometric character and hence identify contiguous clusters in both homogenous and heterogeneous urban tissues. These contextual values are then used as an input for cluster analysis while the original non-contextualised versions are left out, making the final form component composed of 177 contextual characters.

**Table 1:** Morphometric characters used to describe the form component of spatial signatures (part 1).

character	category	reference
area of building	dimension	^41^
perimeter of building	dimension	^42^
courtyard area of building	dimension	^43^
circular compactness of building	shape	^21^
corners of building	shape	^44^
squareness of building	shape	^44^
equivalent rectangular index of building	shape	^45^
elongation of building	shape	^44^
centroid - corner distance deviation of building	shape	^23^
centroid - corner mean distance of building	dimension	^43^
orientation of building	distribution	^43^
street alignment of building	distribution	^43^
cell alignment of building	distribution	^23^
longest axis length of ETC	dimension	^23^
area of ETC	dimension	^13^
circular compactness of ETC	shape	^23^
equivalent rectangular index of ETC	shape	^23^
orientation of ETC	distribution	^23^
covered area ratio of ETC	intensity	^46^
length of street segment	dimension	^12^
width of street profile	dimension	^11^
openness of street profile	distribution	^11^
width deviation of street profile	diversity	^11^
linearity of street segment	shape	^11^
area covered by edge-attached ETCs	dimension	^23^
buildings per meter of street segment	intensity	^23^
area covered by node-attached ETCs	dimension	^23^
alignment of neighbouring buildings	distribution	^47^
mean distance between neighbouring buildings	distribution	^47^

For details of the implementation, refer to the reproducible Jupyter notebooks available at urbangrammarai.xyz.

**Table 2:** Morphometric characters used to describe the form component of spatial signatures (part 2).

character	category	reference
perimeter-weighted neighbours of ETC	distribution	^23^
area covered by neighbouring cells	dimension	^23^
reached ETCs by neighbouring segments	intensity	^23^
reached area by neighbouring segments	dimension	^23^
node degree of junction	distribution	^48^
mean distance to neighbouring nodes of street network	dimension	^23^
mean inter-building distance	distribution	^49^
weighted reached enclosures of ETC	intensity	^23^
reached ETCs by tessellation contiguity	intensity	^23^
reached area by tessellation contiguity	dimension	^23^
area of enclosure	dimension	^21^
perimeter of enclosure	dimension	^12^
circular compactness of enclosure	shape	^43^
equivalent rectangular index of enclosure	shape	^45^
compactness-weighted axis of enclosure	shape	^50^
orientation of enclosure	distribution	^12^
perimeter-weighted neighbours of enclosure	distribution	^23^
area-weighted ETCs of enclosure	intensity	^23^
local meshedness of street network	connectivity	^50^
mean segment length within 3 steps	dimension	^23^
local cul-de-sac length of street network	dimension	^23^
reached area by local street network	dimension	^23^
reached ETCs by local street network	intensity	^23^
local node density of street network	intensity	^23^
local proportion of cul-de-sacs of street network	connectivity	^51^
local proportion of 3-way intersections of street network	connectivity	^48^
local proportion of 4-way intersections of street network	connectivity	^48^
local degree weighted node density of street network	intensity	^21^
local closeness of street network	connectivity	^52^
square clustering of street network	connectivity	^23^

For details of the implementation, refer to the reproducible Jupyter notebooks available at urbangrammarai.xyz.

#### Function

Characterisation of the function component uses a different approach. While data describing urban form are not generally available in a processed format, forcing us to employ morphometric approaches, different aspects of function are often available as open data products. We guide the compilation of functional characters following three main principles: first, we identify from the literature on urban function key areas to be represented; second, we translate those abstract areas into measurable features; and third, we select open data available in for Great Britain that allows for the redistribution of derivative products. With a list of function characters selected, the main goal of our characterisation of ETCs based on function is to develop appropriate transfer methods to link data published as grids or linked to administrative boundaries to ETCs. In this work, we are using five different transfer methods: Areal interpolation, Building-based dasymetric areal interpolation^25^ using building footprint area, Network-constrained accessibility, Euclidean accessibility, and Zonal statistics. Areal interpolation is used when the functional data covers the entirety of space in the form of polygon geometry and when there is no assumption that the phenomena it captures are linked directly to the human population, such as land cover data. When there is an assumption of relation to the population, building-based dasymetric areal interpolation is used instead. The main difference is that instead of ETC polygons, building footprint polygons linked to individual ETCs are used as a target of interpolation. That ensures that data like population estimates are linked to ETCs proportionally to their ability to house population rather than by their area. Network-constrained accessibility is used when the input data represents points of interest like locations of supermarkets. Points are then snapped to the nearest node on the street network and linked to the ETCs through the count of observations accessible from the cell within 15 minutes of walk (1200 m on the street network) and a distance to the nearest point. In some cases, Euclidean (as-crow-flies) accessibility is measured instead to accommodate for phenomena that are often outside the reach of a drivable network like water bodies. Zonal statistics are used to transfer data originally stored in a raster format to ETCs as the mean value of raster pixels intersecting each polygon geometry. Finally, characters based on interpolation and zonal statistics are expressed using their contextual versions following the method used for form characters to, again, reflect the contextual pattern of measured values. As in the case of morphometric characters, only contextual versions are then used in the cluster analysis. The selection of datasets and the chosen transfer method are listed in the Supplementary Table 1.

### Cluster analysis

**Fig. 5:**
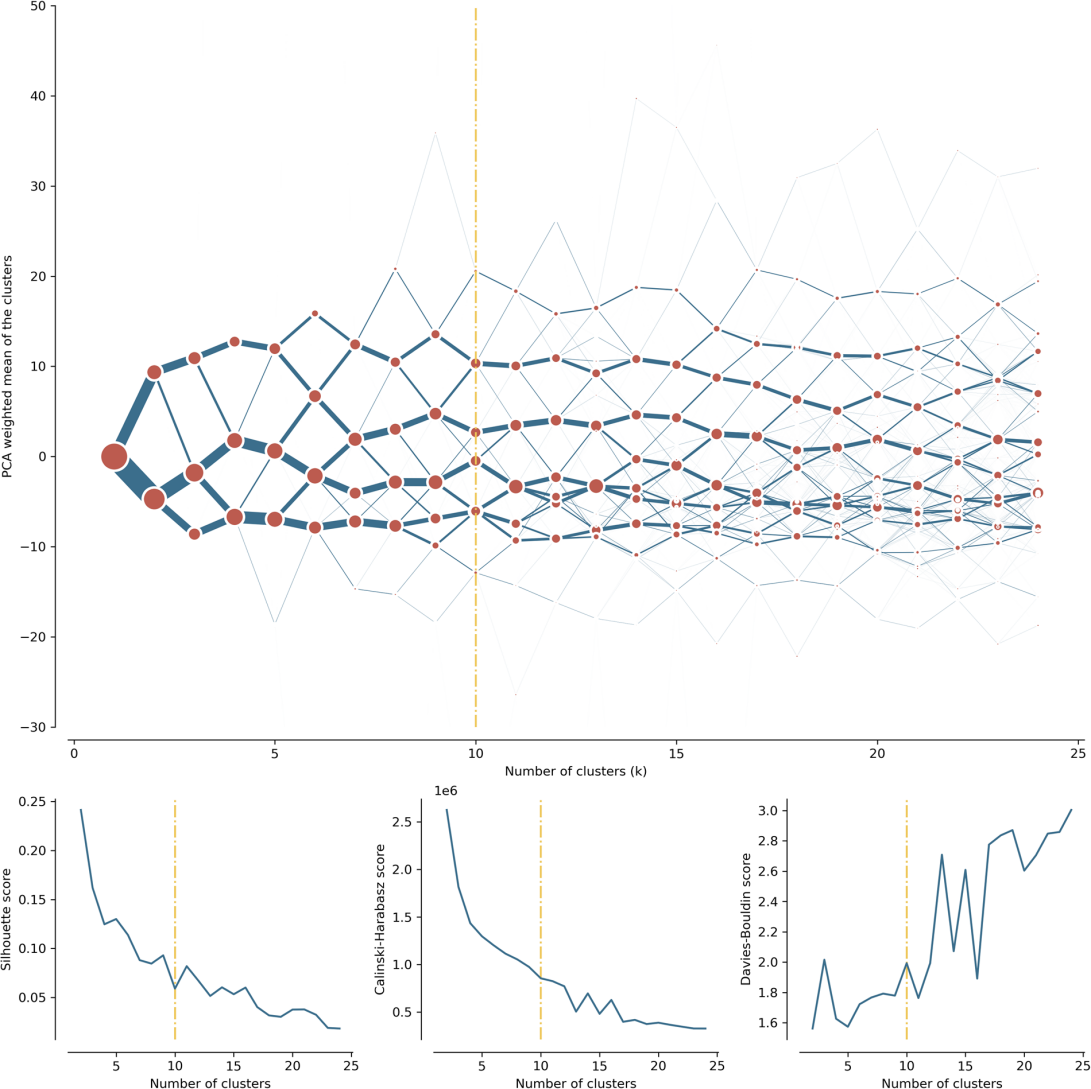
Clustergram and relevant metrics of a goodness of fit (Silhouette score, Calinski-Harabazs score, Davies-Bouldin score) for tested numbers of clusters. The clustergram suggest two potential solutions, the very conservative option of 4 clusters and 10 clusters selected as an optimal result (indicated by a vertical yellow line).

When combined, contextual summaries of form and function characters (or characters themselves when they are reflecting the context by definition) compose a dataset describing each ETC by 328 variables (177 contextual characters representing 59 initial characters for form and 151 for function composed of 144 contextual characters representing 48 characters that do not capture context by design and 10 accessibility-based characters that do). Assigning equal weight to each variable, we standardize them applying Z-score normalization, and use them as input for K-Means cluster analysis. Although collinearity is likely to be present between several of them, we do not view this as a problem: we select each character not from a purely statistical point of view (i.e., which ones will be more effective at segmenting the dataset), but instead from a conceptual one. Each variable has been identified by the literature on urban form and function as a relevant aspect that contributes to collectively characterising these two more abstract concepts. We thus see this situation as a way of adding robustness to the measurement of more conceptual notions which are ultimately our aim. We opt for K-Means because we consider it strikes a compromise in the trade-off between performance and scalability. K-Means is widely used in the literature on unsupervised learning, and in much of that concerning the clustering of geographic entities^26^. To select the algorithm, we experimented with a random subset of our dataset, comparing K-Means with alternatives such as Gaussian Mixture Models (GMM) or Self-Organising Maps (SOM). We found results from the latter two were not notably better in terms of cluster compactness and qualitative examination of the geographic clusters, but were significantly slower in computation runtime, posing serious challenges to be run at scale. Although K-Means does not consider space explicitly, our approach incorporates information about the geographic context of each observation through the operation described above and illustrated in Fig. 4. We prefer this over a spatially-constrained algorithm (e.g., SKATER^27^) that restricts the clustering only among spatially contiguous observations because we are not interested in areas that are spatially contiguous unless they are sufficiently similar to each other on the attribute space. Our contextual approach is more similar to spatially-encouraged algorithms such as the GeoSOM^28^ or spatially-encouraged spectral clustering^29^ that incorporate geographic proximity when clustering but do not restrict. Our choice in this case was led by its scalability over other such algorithms. Nevertheless, we consider this a fruitful avenue for future research. Due to the nature of the selected K-Means clustering, the step preceding the final analysis is the selection of an optimal number of clusters. We use the clustergram exploratory method^30^, reflecting the behaviour of different options, the relationship between clustering solutions regarding the allocation of individual observations to classes, and the separation between the clusters within each tested solution (Fig. 5). Clustergram is further accompanied by measures of internal validation measures - the Silhouette score diagram, Calinski-Harabasz index^31^ and Davies-Bouldin index^32^. The optimal number of classes is selected based on the interpretation of clustergram supported by additional measures aiming at a balance between cluster separation and an appropriate detail of resulting classification. We use mini batch K-Means with a batch size of 1,000,000 and 100 initialisations to create the clustergram and test number of clusters between 2 and 25. The results indicate 10 clusters as an optimal solution. The final clustering solution is generated using mini batch K-Means with a batch size of 1,000,000 and 1,000 initialisations to ensure the stability of the outcome. The results of the clustering capture the first group of a national signature classification composed of ten clusters. However, since the classified ETCs cover the entirety of space, from vast natural open spaces to dense city centres, it may result in only a few classes representing urban areas. While that is caused by the variable heterogeneity of our dataset in combination with K-Means clustering, the measured characters have the ability to further distinguish classes of already identified clusters. As spatial signatures are focused on the urban environment, we further subdivide those clusters covering a substantial portion of urban areas using another iteration of K-Means clustering (one class into nine and another into three clusters). Both subdivisions were created using standard K-Means (single batch) using 1,000 initialisations. The resulting classification then provides a classification capturing the typology of spatial signatures with a detailed focus on urban development. Finally, individual spatial signature geometries are generated as a combination of adjacent ETCs belonging to the same signature class. To describe each geometry and each signature type, we measure mean values of the original, non-contextualised characters, and release it as additional descriptive tables. The resulting numerical profile of each signature type is available as a Supplementary Table 2. Tables 3 and 4 contain pen portraits derived from these numerical profiles.

**Table 3:** Interpretative pen portraits characterising each signature type based on its numerical profile (part 1).

Signature type	Pen Portait
Wild countryside	In “Wild countryside”, human influence is the least intensive. This signature covers large open spaces in the countryside where no urbanisation happens apart from occasional roads, cottages, and pastures. You can find it across the Scottish Highlands, numerous national parks such as Lake District, or in the majority of Wales.
Countryside agriculture	“Countryside agriculture” features much of the English countryside and displays a high degree of agriculture including both fields and pastures. There are a few buildings scattered across the area but, for the most part, it is green space.
Urban buffer	“Urban buffer” can be characterised as a green belt around cities. This signature includes mostly agricultural land in the immediate adjacency of towns and cities, often including edge development. It still feels more like countryside than urban, but these signatures are much smaller compared to other countryside types.
Open sprawl	“Open sprawl” represents the transition between countryside and urbanised land. It is located in the outskirts of cities or around smaller towns and is typically made up of large open space areas intertwined with different kinds of human development, from highways to smaller neighbourhoods.
Disconnected suburbia	“Disconnected suburbia” includes residential developments in the outskirts of cities or even towns and villages with convoluted, disconnected street networks, low built-up and population densities, and lack of jobs and services. This signature type is entirely car-dependent.
Accessible suburbia	“Accessible suburbia” covers residential development on the urban periphery with a relatively legible and connected street network, albeit less so than other more urban signature types. Areas in this signature feature low density, both in terms of population and built-up area, lack of jobs and services. For these reasons, “accessible suburbia” largely acts as dormitories.
Warehouse/Park land	“Warehouse/Park land” covers predominantly industrial areas and other work-related developments made of box-like buildings with large footprints. It contains many jobs of manual nature such as manufacturing or construction, and very little population live here compared to the rest of urban areas. Occasionally this type also covers areas of parks with large scale green open areas.
Gridded residential quarters	“Gridded residential quarters” are areas with street networks forming a well-connected grid-like (high density of 4-way intersections) pattern, resulting in places with smaller blocks and higher granularity. This signature is mostly residential but includes some services and jobs, and it tends to be located away from city centres.

**Table 4:** Interpretative pen portraits characterising each signature type based on its numerical profile (part 2).

Signature type	Pen Portait
Connected residential neighbourhoods	“Connected residential neighbourhoods” are relatively dense urban areas, both in terms of population and built-up area, that tend to be formed around well-connected street networks. They have access to services and some jobs but may be further away from city centres leading to higher dependency on cars and public transport for their residents.
Dense residential neighbourhoods	A “dense residential neighbourhood” is an abundant signature often covering large parts of cities outside of their centres. It has primarily residential purpose and high population density, varied street network patterns, and some services and jobs but not in high intensity.
Dense urban neighbourhoods	“Dense urban neighbourhoods” are areas of inner-city with high population and built-up density of a predominantly residential nature but with direct access to jobs and services. This signature type tends to be relatively walkable and, in the case of some towns, may even form their centres.
Local urbanity	“Local urbanity” reflects town centres, outer parts of city centres or even district centres. In all cases, this signature is very much urban in essence, combining high population and built-up density, access to amenities and jobs. Yet, it is on the lower end of the hierarchy of signature types denoting urban centres with only a local significance.
Regional urbanity	“Regional urbanity” captures centres of mid-size cities with regional importance such as Liverpool, Plymouth or Newcastle upon Tyne. It is often encircled by “Local urbanity” signatures and can form outer rings of city centres in large cities. It features high population density, as well as a high number of jobs and amenities within walkable distance.
Metropolitan urbanity	Signature type “Metropolitan urbanity” captures the centre of the largest cities in Great Britain such as Glasgow, Birmingham or Manchester. It is characterised by a very high number of jobs in the area, high built-up density and often high population density. This type serves as the core centre of the entire metropolitan areas.
Concentrated urbanity	Concentrated urbanity” is a signature type found in the city centre of London and nowhere else in Great Britain. It reflects the uniqueness of London in the British context with an extremely high number of jobs and amenities located nearby, as well as high built-up and population densities. Buildings in this signature are large and tightly packed, forming complex shapes with courtyards and little green space.
Hyper concentrated urbanity	The epitome of urbanity in the British context. “Hyper concentrated urbanity” is a signature type present only in the centre of London, around the Soho district, and covering Oxford and Regent streets. This signature is the result of centuries of urban primacy, with a multitude of historical layers interwoven, very high built-up and population density, and extreme abundance of amenities, services and jobs.

## Data Records

The data product^33^ described in this article is available through the Consumer Data Research Centre Open Data repository^34^ under the Open Government Licence v3.0 license and archived^33^. The dataset stored in the repository contains a GeoPackage with a signature geometry (OSGB36/British National Grid (EPSG:27700) CRS) and related signature type, plain-text pen portraits describing individual signature types, a series of CSV files describing individual signatures and signature types, and a CSV files linking signature types to the Output Area and Lower Super Output Area geometry. An online interactive map of spatial signatures for the whole of Great Britain is available on the project website (https://urbangrammarai.xyz/great-britain). The underlying data used to create the ODP are available in a dedicated GitHub repository available from (https://github.com/urbangrammarai/signatures_gb).

## Technical Validation

### Character importance

The characters used in the cluster analysis have each different importance in distinguish between signature types. Those characters which spatial distribution most closely matches the distribution of signatures can be seen as more important that those that are seemingly random or mostly invariant (as some of the land cover classes are). Unpacking the importance of individual characters from K-Means clustering cannot be done directly. However, we provide indirect evidence from two different approaches. First, we can use the F-test to assess the significance of the relationship between characters and signature types by regressing each character on a set of indicator variables with our signature classes. If the variation in the character maps onto that between classes, the F-test will reject the null hypothesis and will be considered significant. In the second exercise, we train a supervised model, in our case Random Forest, designed to predict individual signature types from input data. The former unpacks whether all the characters play a role in the delineation of clusters while the latter provides indication on feature importance - a relative measure of strength of each character in distinguishing between the types. Out of 328 characters, 18 are invariant (the full list includes: ‘Land cover [Airports] Q1, Land cover [Mineral extraction sites] Q1, Land cover [Road and rail networks and associated land] Q1, Land cover [Water bodies] Q1, Land cover [Inland marshes] Q1, Land cover [Dump sites] Q1, Land cover [Water courses] Q2, Land cover [Burnt areas] Q2, Land cover [Water courses] Q1, Land cover [Burnt areas] Q1, Land cover [Agro-forestry areas] Q3, Land cover [Coastal lagoons] Q2, Land cover [Burnt areas] Q3, Land cover [Agro-forestry areas] Q1, Land cover [Agro-forestry areas] Q2, Land cover [Dump sites] Q2, Land cover [Coastal lagoons] Q1, Land cover [Coastal lagoons] Q3’) and five insignificant at the 5% level (the full list includes: Land cover [Green urban areas] Q1, Land cover [Road and rail networks and associated land] Q2, Land cover [Water bodies] Q2, Land cover [Transitional woodland-shrub] Q1, Land cover [Coniferous forest] Q1) (all derived from land cover) according to the F-test results. The results of the Random Forest-based feature importance approach are shown in a Table 5. As can be seen, form-based characters dominate the top 10 characters, but it is worth noting that these top 10 characters together bear only 0.196 of the overall importance. A similar exercise can be done on at the level of individual clusters, with a binary Random Forest model trained to distinguish that particular class from the other. Resulting relative importance of top 10 characters for each signature type is presented in a Supplementary Table 3. While it is clear that form-based characters still dominate the prediction, the more urban signature types are, the higher the importance of function seems to be. Complete tables with all characters are available as online Tables 1 and 2.

**Table 5:** Relative importance of top 10 most important characters in predicting spatial signature types using the Random Forest model.

	relative importance
covered area ratio of ETC (Q1)	0.036944
covered area ratio of ETC (Q2)	0.031717
perimeter-weighted neighbours of ETC (Q2)	0.023476
mean inter-building distance (Q2)	0.016662
area of ETC (Q3)	0.016005
area covered by node-attached ETCs (Q3)	0.014813
longest axis length of ETC (Q2)	0.014501
weighted reached enclosures of ETC (Q1)	0.014115
reached area by neighbouring segments (Q3)	0.014000
reached area by neighbouring segments (Q1)	0.013904

### Comparison

Spatial signatures are unique as a classification method, limiting the potential validation. Therefore, we rather present a comparison of signatures and ancillary datasets capturing conceptually similar aspects of the environment. We compare the signatures with four of such datasets, each focusing on a different classification perspective, but all related to our classification to a degree when we can assume there will be a measurable level of association between the two:

- WorldPop settlement patterns of building footprints (2021)^10^
- Classification of Multidimensional Open Data of Urban Morphology (MODUM) (2015)^7^
- Copernicus Urban Atlas (2018)^35^
- Local Climate Zones (2019)^36^

### Comparison approach

All datasets, spatial signatures and those selected for a comparison contain a categorical classification of space linked to their unique geometry. The first requirement to be able to compare data products is to transfer their information to the same geometry. We take two approaches for this step, depending on the dataset we are comparing the signatures with: an interpolation of one set of polygon-based data to another (input to ETCs); or the conversion of spatial signatures to the raster representation matching an input raster, which is computationally more efficient when one of the layers is already a raster. The second step is a statistical comparison of two sets of classification labels, one representing spatial signature typology and the other comparison classes. We use contingency tables and Pearson’s *χ*^2^ test to determine whether the frequencies of observed (signature types) and expected (comparison types) labels significantly differ in one or more categories. Furthermore, we use Cramér’s *V* statistics^37^ to assess the strength of the association.

### WorldPop settlement patterns of building footprints

**Fig. 6:**
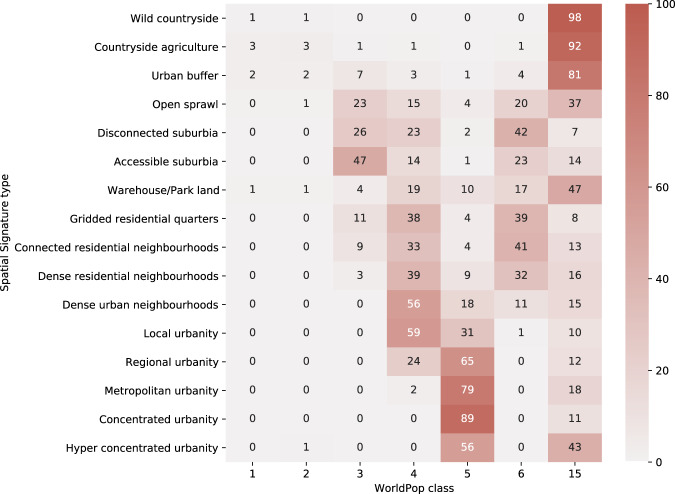
Contingency table showing frequencies (in %) of WorldPop classes within signature types.

WorldPop settlement patterns of building footprints dataset aims to derive a typology of morphological patterns based on a gridded approach with cells of 100 × 100 m, and building footprints. Authors measure six morphometric characters linked to the grid cells and use them as input for an unsupervised clustering algorithm leading to a six-class typology. As the classification is dependent on building footprints, grid cells that do not contain any information on the building-based pattern are treated as missing in the final data product. For the comparison, this *missing* category is treated as a single class. It is assumed that the top-level large scale patterns detected by the WorldPop method and spatial signatures will provide similar results. However, there will be differences caused by the inclusion of function in spatial signatures, higher granularity of both initial spatial units and the resulting classification (6 vs 19 classes). Signature typology is rasterized and linked to the WorldPop grid. The resulting contingency table is shown in Fig. 6. There is a significant relationship between two typologies, *χ*^2^ (114, *N* = 22993921) = 13341832, *p* < 0.001. The strength of association measured as Cramér’s *V* is 0.311, indicating moderate association. The contingency table shows that WorldPop classes tend to be linked to groups of signature types of a similarly degree of urbanity. A WorldPop class 15 is “undefined” due to the lack of building footprints in the area, therefore overlapping a large portion of signatures. The difference between classifications is likely driven by two main aspects - one is the different number of classes. We can see that WorldPop classes tend to cluster within a limited number of signature types and vice versa. The only exception is allocation of signature types into classes 4 and 6, which seems to heavily overlap. That is possibly caused by the second aspect - inclusion of function. Both classes 4 and 6 tend to be outside of city centres but still within urban areas. While it is the footprint-based form that is driving the difference between them, signatures in the same area are often distinguished by function and varies access to amenities and services.

### Multidimensional open data urban morphology

**Fig. 7:**
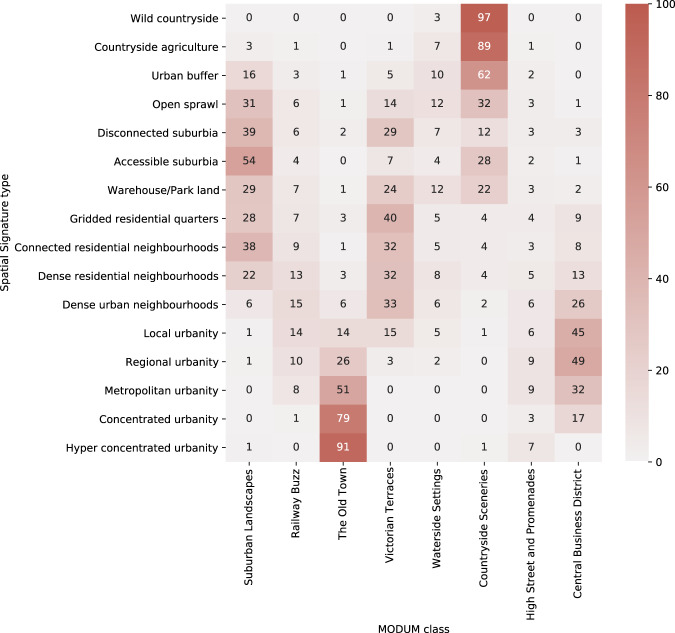
Contingency table showing frequencies (in %) of MODUM classes within signature types.

Multidimensional Open Data Urban Morphology (MODUM) classification describes a typology of neighbourhoods derived from 18 indicators capturing built environment as streets, railways or parks, linked to the Census Output Area geometry. The classification identifies 8 types of neighbourhoods. Compared to the WorldPop classification, MODUM takes into account more features of the built environment than building footprints, which makes it conceptually closer to the spatial signatures. However, it is still focusing predominantly on the form component, although there are some indicators that would be classified as function within the signatures framework (e.g. population). The MODUM method uses a different way of capturing context compared to the signatures, which leads to some classes being determined predominantly by a single character. For example, the *Railway Buzz* type forms a narrow strip around the railway network, which is an effect signatures avoid. MODUM typology is available only for England and Wales. Therefore, the comparison takes into account only ETCs covering the same area. The classification is linked to the ETC geometry is based on the proportion (the type covering the largest portion of ETC is assigned). The resulting contingency table is shown in Fig. 7. There is a significant relationship between two typologies, *χ*^2^ (152, *N* = 13067584) = 13938867, *p* < 0.001. The strength of association measured as Cramér’s *V* is 0.300, indicating moderate association of very similar levels we have seen above. The contingency table indicates similar relationships, where a single MODUM class overlaps a group of signature types. However, the groups tend to be well defined and formed based on the similarity of types. Signature types are minimally present in MODUM classes driven by a single character (*Railway Buzz*, *Waterside Settings*, *High Street and Promenades*), suggesting the more balanced weight of characters.

### Copernicus urban atlas

**Fig. 8:**
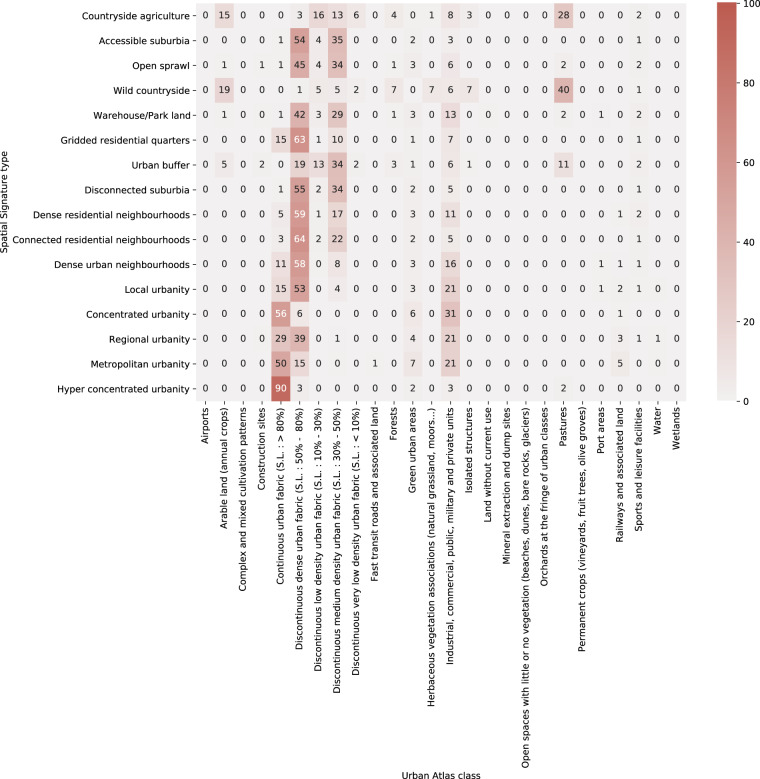
Contingency table showing frequencies (in %) of Urban Atlas classes within signature types.

Copernicus Urban Atlas is the least similar of the comparison datasets. It is a high-resolution land use classification of functional urban areas derived primarily from Earth Observation data enriched by other reference data as OpenStreetMap or topographic maps. Its smallest spatial unit in urban areas is 0.25 ha and 1 ha in rural areas, defined primarily by physical barriers. It identifies 27 predefined classes using the supervised method. The majority of urban areas is classified as urban fabric further distinguished based on continuity and density resulting in six classes of the urban fabric. The classification does not consider the type of the pattern or any other aspect. Furthermore, it does not take into account what signatures call *context* as each spatial unit is classified independently, which in some cases leads to the high heterogeneity of classification within a small portion of land. Signatures take a different approach. Consequently, it is expected that the similarity between the two will be limited. Urban Atlas is available only for functional urban areas (FUA), leaving rural areas unclassified. Comparison then applies to FUAs only. The classification is linked to the ETC geometry based on the proportion (the type covering the largest portion of ETC is assigned). The resulting contingency table is shown in Fig. 8. There is a significant relationship between two typologies, *χ*^2^ (450, *N* = 8396642) = 5229900, *p* < 0.001. The strength of association measured as Cramér’s *V* is 0.186, indicating a weak association. The contingency table shows the difference in the aim of spatial signatures and that of Urban Atlas with a majority of signatures being linked to a few of Urban Atlas classes. Within relevant classes, we see a tendency of signature types to cluster within Urban Atlas classes based on the level of urbanity, albeit not as strong as in the previous two cases. The main reason behind such a large difference are the aims of both classifications. While the Copernicus Urban Atlas attempts to capture land cover, resulting in a large number of non-urban classes, spatial signatures are aimed at urban environment with 13 out of 16 classes covering primarily urbanised areas.

### Local climate zones

**Fig. 9:**
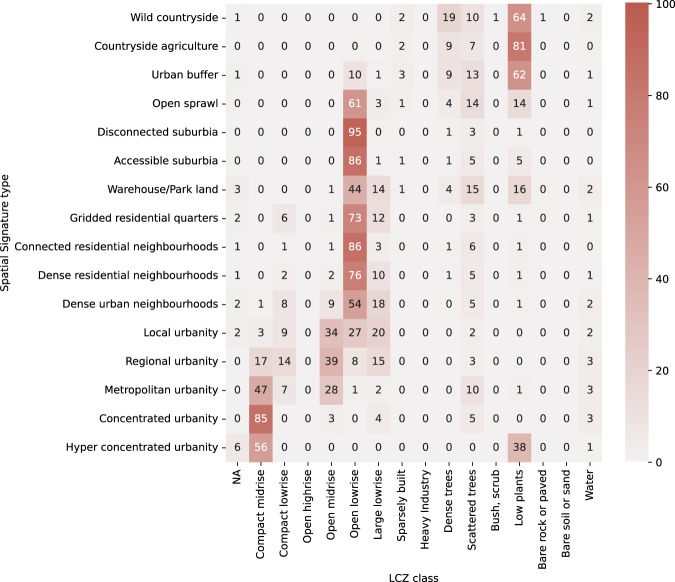
Contingency table showing frequencies (in %) of Local Climate Zones within signature types.

Local climate zones (LCZ) are conceptual classes originally designed to support study of urban climate as temperature. It consists of 17 classes of which 10 can be classified as urban and 7 and natural ones. In the context of Great Britain, the dataset used in this study does not contain 2 of them, *Lightweight low-rise* and *Compact highrise* as they are not present in the British landscape. The datasets produced by^36^ released LCZs in a 100 meters grid based on the 2016 data. As the LCZs are remotely sensed in this case, authors report overall average accuracy of 80% As a conceptual classification aimed to cover all possible types of primarily urban climate zones globally, LZCs may not be optimal when looking into a single country with specific history of urban development. This is further indicated by classes that are missing. It is therefore likely that large parts of British cities will fall into only a few of LCZ classes, while being represented by a much larger number of signature types. Signature typology is rasterized and linked to the LCZ grid. The resulting contingency table is shown in Fig. 9. There is a significant relationship between two typologies, *χ*^2^ (225, *N* = 16203338) = 18467242, *p* < 0.001. The strength of association measured as Cramér’s *V* is 0.276, indicating a modest to weak association, close to values we have seen in first two cases. As expected, urban signature types are clustered primarily within *Compact midrise* and *Open lowrise* LCZs, while non-urban signatures mostly fall into the *Low plants* LCZ. The difference between signatures and LCZs can be accounted to two aspects. One, as we have seen before is the inclusion of function in spatial signatures, differentiating e.g. LCZ’s *Open lowrise* into many signature types. The other is data-driven nature of signatures compared to conceptual LCZs, where differences in signature types are below the resolution capability of simple matrix composed of density and compactness levels. On the other, it is encouraging to see that most of signature types fall predominantly in a single LCZ class, suggesting that while both classifications are built differently, they are able to capture similar large-scale patterns in cities.

### Summary

None of the comparisons shows more than a moderate association, but since none of the comparison datasets is aiming to capture the same conceptualization of space as spatial signatures do, such a result is expected. The moderate association with both WorldPop settlements patterns and MODUM is reassuring as both are conceptually closer to signatures than the Urban Atlas (especially in their unsupervised design). Urban Atlas, though very different in its aims and methods, still shows a measurable association, which we interpret as sign that the key structural aspects forming cities are captured by both. The comparison exercise suggests that general patterns forming cities are shared among signatures and existing typologies. Signature types tend to form groups when we look at their relation to comparison classes and it is not uncommon that a single signature type is present in multiple groups linked to different classes. However, all these groups tend to be formed based on the similarity and illustrate the granularity of the presented classification compared to existing datasets, allowing us to distinguish, for example, five types of signature types forming town and city centres.

## Usage Notes

The released data product follows widespread standards for geographic data storage and should be easy to integrate with other data and methods by researchers wanting to reuse it. However, due to the density of signature geometry (resulting from the detailed ETCs), it may be needed to simplify the geometry for a smoother interactive experience on machines with limited resources. Replication of the analysis optimally requires at least a single computational node with a large amount of RAM (+100GB) due to the size of the input data and detail on which signature characterisation is computed. It is also recommended revisiting the state of the development of related software packages, notably momepy^38^, libpysal^39^, tobler^25^ and dask-geopandas as they may soon offer more efficient drop-in replacements of the custom code used to produce this dataset.

### Supplementary information

Supplementary table 2

Supplementary table 3

Supplementary table 1

## Data Availability

The source code used to produce this dataset is openly available in a GitHub repository at https://github.com/urbangrammarai/spatial_signatures and in the form of a website on https://urbangrammarai.xyz. Code is organized in a series of Jupyter notebooks and have been executed within the darribas:gds_dev:6.1^40^ Docker container, unless specified otherwise in the individual notebooks.
