# Author Correction: Geographical characterisation of British urban form and function using the spatial signatures framework

**DOI:** 10.1038/s41597-023-02773-0

**Published:** 2023-12-01

**Authors:** Martin Fleischmann, Daniel Arribas-Bel

**Affiliations:** 1 Geographic Data Science Lab, Department of Geography and Planning, University of Liverpool, Roxby Building, 74 Bedford St S, Liverpool, L69 7ZT UK; 2 The Alan Turing Institute, British Library, 96 Euston Road, London, England NW1 2DB UK

**Keywords:** Geography, Society, Geography, Society

Correction to: *Scientific Data* 10.1038/s41597-022-01640-8, published online 07 September 2022

The original figure 5 was incorrect, due to the wrong file being submitted prior to typesetting, and has now been replaced with the correct version in the HTML and pdf versions of the manuscript. No other details, such as the figure caption, or description within the text, have been changed.

